# Is a History of Malignant Melanoma Associated with Subsequent Vitiligo? Insights from a Population-Based Case–Control Study

**DOI:** 10.3390/jcm14155546

**Published:** 2025-08-06

**Authors:** Talia Israel, Baruch Kaplan, Naama T. Cohen, Shany Sherman, Geffen Kleinstern, Khalaf Kridin

**Affiliations:** 1 School of Public Health, Faculty of Social Welfare and Health Sciences, University of Haifa, Haifa 3498838, Israelgkleinste@univ.haifa.ac.il (G.K.); 2 Adelson School of Medicine, Ariel University, Ariel 40700, Israel; 3 Azrieli Faculty of Medicine, Bar-Ilan University, Safed 1311502, Israel; 4 Division of Dermatology, Rabin Medical Center—Beilinson Hospital, Petach Tikva 4941492, Israel; shanyshnush@gmail.com; 5 Faculty of Medicine, Tel Aviv University, Tel Aviv 6997801, Israel; 6 Unit of Dermatology and Skin Research Laboratory, Galilee Medical Center, Nahariya 2210001, Israel; 7 Lübeck Institute of Experimental Dermatology, University of Lübeck, 23562 Lübeck, Germany

**Keywords:** malignant melanoma, vitiligo, case–control study, epidemiology

## Abstract

**Background**: While a few studies suggest that depigmentation tends to develop more frequently in patients with malignant melanoma (MM), the association between vitiligo and MM has been sparsely investigated in the setting of controlled studies. **Methods**: A population-based case–control study compared 14,632 patients with vitiligo with 71,580 control subjects matched by age, sex, and ethnicity regarding the prevalence of preexisting MM. Logistic regression was used to calculate the odds ratio (OR) and 95% confidence intervals (CIs) of developing vitiligo both in univariate and multivariate models, adjusting for demographic variables and comorbidities. The OR was also stratified by age, sex, ethnicity, and ultraorthodox status. **Results**: The prevalence of preexisting MM was statistically comparable between individuals with vitiligo and controls (0.30% vs. 0.35%, respectively). In the general study population, a history of MM was not significantly associated with an elevated likelihood of developing vitiligo (multivariate OR, 1.03; CI 95%, 0.76–1.40). Among the Arab population, however, preexisting MM was associated with a sixfold-increased likelihood of subsequent vitiligo (univariate OR, 6.55; 95% CI, 1.46–29.27). Patients with vitiligo and comorbid MM were older at the onset of vitiligo, had a higher burden of comorbid conditions, and showed an overrepresentation of Jewish ancestry. **Conclusions**: A history of MM does not increase the probability of vitiligo in the general Israeli population, except among the Arab minority, who show a sixfold-elevated odds of vitiligo after MM. Further investigation is essential to gain deeper insights into this relationship.

## 1. Introduction

Malignant melanoma (MM) represents the uncontrolled growth of melanocytes in the form of melanoma [1] and is one of the most fatal types of skin cancer at advanced stages of the disease [2]. The disease occurs in 1.6% of newly diagnosed skin cancers and causes 0.7% of deaths from all cases of cancer annually [3]. The highest incidences and mortalities are found in Australia and New Zealand, whereas the lowest are in South-Eastern Asia and South-Central Asia [3]. Israel stands in 23rd place, with an incidence rate of 0.01%, based on Israel’s population size of 9,140,500 people at the end of 2020 [4], and varies between Jews and Arabs, where 98.6% of MM cases are Jews and others, and only 1.4% are Arabs [5].

The major risk factor for MM is exposure to ultraviolet radiation by sun exposure, sunburns, or indoor tanning (especially for patients aged ≤ 35 years old), which may cause alterations in DNA [1]. Other risk factors for MM are a personal or family history of cutaneous MM; phenotypic characteristics including fair hair, eye, and skin colors; a tendency to freckle; and high socioeconomic status [1]. About 50% of cutaneous MM is due to activation of oncogene signal transduction mutation at the BRAF, which causes early-stage MM [6]. More invasive MM requires more mutations in the gene code and activation of other oncogenes [1].

Vitiligo is a chronic and acquired autoimmune disease, with a prevalence ranging between 0.1% to 2% in the general population, including children and adults [7,8], that is characterized by depigmented macules and patches on the skin, hair, and mucosae that appear due to the destruction of melanocytes [9]. Vitiligo is typified by a strong association with other autoimmune diseases such as type-1 diabetes, rheumatic arthritis, Addison’s disease, and autoimmune thyroiditis [10]. Multiple lines of evidence suggest that a combination of genetic, environmental, and autoimmune triggers could initiate the disease [11,12,13].

Melanocytes produce the pigment melanin, the main component that provides color to the skin and absorbs ultraviolet light that could damage DNA in the skin cells. People with darker skin produce melanin from eumelanin, while people with lighter skin produce melanin from pheomelanin. In addition, darker skin is characterized by a larger amount of melanin, while lighter skin is characterized by a lower amount of melanin [14]. The risk for developing MM varies among races and ethnicities, with Caucasian people at a higher risk of developing MM than black people [15]. While vitiligo is more notable in people with darker skin phototypes, the differences between ethnic groups are controversial. Recent studies have found that vitiligo is more common in Asian, Black, and Hispanic/Latino patients than white patients [16,17,18]. Other studies did not reveal significant differences among different races [19,20].

The biological connection between MM and vitiligo is incompletely understood. However, a few studies suggest that depigmentation tends to develop more frequently in patients with MM [21,22,23]. It can occur spontaneously before or after the diagnosis of MM or as a response to immunotherapy medications [24]. Antibodies that target tumor cells may also attack normal melanocytes, impairing their function and leading to the phenomenon known as ‘melanoma-associated vitiligo’ (MAV) [22,23]. Interestingly, the appearance of MAV in MM patients has been associated with a better prognosis, suggesting a stronger immune response against both the tumor and normal melanocytes [23]. MAV is believed to be a parallel but distinct condition from vitiligo [24]. The prevalence of MAV and vitiligo in the population of patients suffering from MM is higher than that of vitiligo in the general population, ranging between 2 and 16% [23].

In this study, we aim to evaluate whether a prior history of MM impacts the odds of developing vitiligo. The secondary endpoint of our study is to explore whether patients with vitiligo and coexistent MM differ from patients with isolated vitiligo.

## 2. Materials and Methods

A case–control study was performed to assess the association between patients with a prior history of MM and the odds of developing vitiligo. This study was approved by an independent review board of Clalit Health Services (CHS) under the Helsinki number 0212-17-COM. Data was retrieved from CHS database, the largest health maintenance organizations (HMO) that provide health services to Israeli residents through Israel’s State Health Insurance Law, 1994. The CHS covers more than 50% of the Israeli population, with over 1000 branches established throughout the country.

The CHS database was screened for incident cases with a diagnostic code of vitiligo (according to the ICD-9 code 709.01) between the years 2002 and 2019. Patients were considered eligible for inclusion if one of the following criteria was fulfilled: (i) a documented diagnosis of vitiligo as registered by a board-certified dermatologist or (ii) a diagnosis of vitiligo in discharge letters from dermatological wards. The diagnosis of MM relied on the CHS registry for chronic diseases. This registry retrieves data from various sources, including hospital and primary care reports, medication purchases, health service utilization, and laboratory and imaging data. All extracted data is thereafter cross-checked manually and validated by the primary care practitioner of each patient.

We enrolled a control group including up to five individuals without vitiligo per each case. Controls were matched based on age, sex, and ethnicity and were recruited on the day in which the corresponding case was diagnosed. The diagnosis of MM was based on its documentation by a board-certified dermatologist or in discharge letters released from dermatological wards (according to the ICD-9 code 172.9).

Outcome measures were adjusted for putative confounding variables like age, sex, ethnicity, ultraorthodox status, body mass index (BMI), and Charlson comorbidity index (CCI) score. The latter is an epidemiologic score enabling assessment of the extent and severity of comorbidities in study participants; it is methodologically valid in extensive data analyses and has been found to predict mortality in a reliable manner [25].

A descriptive analysis of the patients’ demographic data, including age, sex, ethnicity, weight, height, BMI, and CCI scores was performed by presenting the counts and percentages for categorical variables and average, standard deviation (SD), median, and range for continuous variables. The prevalence of preexisting MM was assessed in both groups, and the odds ratio (OR) was calculated. Logistic regression was used to determine whether a prior history of MM increases the odds of developing vitiligo and was reported in a univariate model, followed by two different adjusted models. Model 1 included all variables (age, sex, CCI score, ethnicity, ultraorthodox status, and BMI), and model 2 included all variables except BMI (due to missing data). The analysis was also stratified by age (18–29, 30–59, ≥60), sex (males vs. females), ethnicity (Jews vs. Arabs), and ultraorthodox status (ultraorthodox vs. others). The case–control design and the presence of temporal sequence between exposure and outcome enabled the estimation of the odds of vitiligo imposed by preceding MM. This was based on the “rare disease assumption” hypothesizing that OR approaches relative risk when investigating rare diseases with a prevalence rate lower than 10% [26].

Secondly, to compare patients with vitiligo and comorbid MM to the rest of the vitiligo patients, a Student’s *t*-test was used for numerical variables, and Chi-squared or Fisher’s exact tests were used for categorical variables. All statistical analyses were conducted using SPSS, version 27.0 (SPSS Inc., Chicago, IL, USA).

## 3. Results

### 3.1. Characteristics of the Study Population

The study population encompassed 86,212 patients—14,632 patients with vitiligo and 71,580 matched control individuals. The mean (SD) age at diagnosis and recruitment of control subjects was 45.6 (17.3) years. Seven thousand three hundred and thirty-five (50.1%) patients with vitiligo were males. The ethnic composition of both groups was statistically comparable, with 78% Jews and 22% Arabs. Patients with vitiligo were found to have a lower mean BMI (26.2 [5.2] vs. 26.6 [5.5], *p* < 0.001), whereas the remaining baseline characteristics were comparable between study groups. Table 1 further presents the full attributes of study participants.

### 3.2. The Odds of Developing Vitiligo Following a History of Malignant Melanoma

The prevalence of preexisting MM was comparable between patients with vitiligo and control individuals (0.30% vs. 0.35%, respectively). This indicates that there was no significant association between a prior history of MM and the likelihood of developing subsequent vitiligo (univariate OR, 0.97; CI 95% 0.72–1.32). A lack of significant association also persisted after adjusting for multiple confounders (multivariate OR, 1.03; CI 95%, 0.76–1.40; Table 2).

Stratification by demographic variables demonstrated that a history of MM was significantly associated with vitiligo only among Arabs, with only 4 out of 3216 Arab patients in the vitiligo group compared to 3 out of 15,779 patients in the control group who had melanoma prior to vitiligo (univariate OR, 6.55; 95% CI, 1.46–29.27). This association was shown to be even stronger after adjusting for putative confounders in the fully adjusted multivariate model (multivariate OR, 9.37; 95% CI, 1.71–51.29). The association was only of marginal statistical significance among ultraorthodox patients, with only 4 out of 418 patients in the vitiligo group compared to 6 out of 2061 patients in the control group who had melanoma prior to vitiligo (multivariate OR, 3.23; 95% CI, 0.90–11.66).

### 3.3. Demographic Characteristics of Patients with Vitiligo and Comorbid MM

The last endpoint of the study was to compare the epidemiological characteristics of patients with vitiligo and comorbid MM (N = 70) relative to the remaining patients with vitiligo (N = 14,562; Table 3). Patients with vitiligo and comorbid MM were significantly older at the onset of vitiligo (61.8 [16.3] vs. 45.6 [17.3] years, respectively; *p* < 0.001), had a higher median CCI score (3.0 vs. 0.7, respectively; *p* < 0.001), and had a prominent majority of Jews (92.8% vs. 77.8%, respectively; *p* = 0.003). The sex distribution and BMI were comparable between the two subgroups (Table 3).

## 4. Discussion

In the current large-scale population-based case–control study, a history of MM was not significantly associated with an elevated risk of vitiligo. One exception was observed among the Israeli Arab population, where preexisting MM predicted a sixfold-heightened odds of vitiligo. Patients with vitiligo and comorbid MM were characterized by an older age of presentation with vitiligo and greater comorbidity burden.

The pathogenesis of vitiligo following malignant melanoma (MM) remains incompletely understood, warranting further investigation. CD8⁺ T cells are known to play a central role, targeting both MM cells and normal melanocytes [27]. A genome-wide association study identified a polymorphism in the TYR gene, which encodes tyrosinase—a key enzyme in melanin synthesis and MM initiation. Notably, major alleles of TYR-related signal nucleotide polymorphisms (SNPs) are associated with vitiligo, while minor alleles are linked to MM [12], suggesting an inverse genetic relationship [11], as antityrosinase expression might protect patients with vitiligo against MM [23]. Additional loci exhibiting this inverse association include RALY-EIF252-ASIPAHCY-ITCH, IRF4, MC1R, HLA-DRB1/DQA1, CTLA4, and PTPN22. Inherited variants in these pigmentation-related genes may contribute to increased MM susceptibility [28].

The elevated odds of vitiligo following MM among the Arab population represent a highly intriguing observation that had not been reported elsewhere. This finding is particularly notable given that MM is relatively rare among Arabs in Israel [5]. Further genetic studies are warranted to shed more light on potential explanations for this epidemiological finding.

Another objective of the study was to investigate whether patients with vitiligo and comorbid MM exhibited distinct characteristics compared to the remaining patients with vitiligo. Patients with comorbid MM featured an older age of presentation (with vitiligo) and higher comorbid burden (as reflected by higher CCI). These findings can be perceived based on the fact that MM typically presents in older individuals [5] and the CCI increases with age due to the accumulation of comorbid conditions [25].

One of the main strengths of the current study is its large-scale study population. The study relied on the well-established CHS database, which includes a broad and diverse patient population from various geographic locations and demographic backgrounds. The large sample size improved the precision of the study estimates and increased the reliability of the results. The ICD-9 coding system provided a standardized and reliable system for diagnosing vitiligo and MM, ensuring consistency across patient records and uniformity in data collection across CHS branches, contributing to the dataset’s consistency and accuracy.

Our study has important limitations that must be acknowledged and cannot be overlooked. A lack of data on MM treatments might embody a potential bias. This is particularly relevant as immunotherapy, which alters the immune system, may affect vitiligo development and could, therefore, impact the results of our study. Residual confounding cannot be thoroughly excluded, as we were unable to adjust for missing variables such as occupation and Fitzpatrick skin type. Similarly, this study did not distinguish between the different stages of MM, which could be a mediator for the treatments patients received. Given its population-based nature, the study lacked documentation of the skin phototype of eligible patients, which is an essential determinant in the development of MM. Additionally, the number of Arab vitiligo participants with a preexisting diagnosis of melanoma was low, which may have contributed to wide confidence intervals and reduced the precision of our estimates.

In conclusion, the current large-scale population-based case–control study revealed that a history of MM does not predispose Israeli individuals to developing subsequent vitiligo, except for the Arab minority, where MM was associated with a sixfold-elevated risk of vitiligo. Further genetic investigation is necessary to account for this intriguing ethnic observation.

## Figures and Tables

**Table 1:** Baseline characteristics of study participants.

	Vitiligo (N = 14,632) N (%)	Controls (N = 71,580) N (%)	*p* Value
Age, years * Mean (SD)	45.7 (17.3)	45.6 (17.2)	0.43
18–29	3410 (23.3)	16,751 (23.4)	
30–59	7671 (52.4)	37,742 (52.7)	
≥60	3551 (24.3)	17,087 (23.9)	0.59
Sex			
Males	7335 (50.1)	35,848 (50)	0.91
Ethnicity			
Jews	11,416 (78)	55,801 (78)	
Arabs	3216 (22)	15,779 (22)	0.86
Ultraorthodox status			
Ultraorthodox	418 (2.9)	2061 (2.9)	
Others	14,214 (97.1)	69,519 (97.1)	0.88
BMI (kg/m^2^) Mean (SD)	26.2 (5.2)	26.6 (5.5)	**<0.001**
Missing	1762 (12)	10,660 (15)	

* Age at the first date of follow-up. Abbreviations: BMI, body mass index; SD, standard deviation. **Bold**: significant values.

**Table 2:** The association between a history of malignant melanoma and subsequent vitiligo stratified by age, sex, ethnicity, and ultraorthodox status.

	Vitiligo (N = 14,632) N (%)	Controls (N = 71,580) N (%)	Univariate OR	CI 95%	Model 1 * Multivariate OR	CI 95%	Model 2 ** Multivariate OR	CI 95%
Overall	50 (0.30)	252 (0.35)	0.97	0.72–1.31	1.03	0.76–1.40	0.99	0.73–1.34
Age, years								
18–29	3 (0.09)	8 (0.05)	1.84	0.49–6.95	1.64	0.42–6.41	1.56	0.41–5.95
30–59	15 (0.19)	75 (0.20)	0.98	0.56–1.71	1.08	0.61–1.89	0.95	0.55–1.66
≥60	32 (0.90)	169 (0.99)	0.91	0.62–1.33	0.98	0.67–1.43	0.97	0.66–1.42
Sex								
Females	22 (0.30)	127 (0.40)	0.87	0.55–1.37	0.87	0.55–1.37	1.13	0.75–1.71
Males	28 (0.40)	125 (0.30)	1.10	0.73–1.65	1.20	0.79–1.81	0.84	0.53–1.33
Ultraorthodox status								
Ultraorthodox	4 (1.00)	6 (0.30)	3.31	0.93–11.78	3.23	0.90–11.66	3.36	0.93–12.08
Others	46 (0.30)	246 (0.40)	0.91	0.67–1.25	0.975	0.71–1.34	0.93	0.68–1.28
Ethnicity								
Jews	46 (0.40)	249 (0.40)	0.90	0.66–1.24	0.96	0.70–1.33	0.93	0.67–1.27
Arabs	4 (0.10)	3 (0.00)	**6.55**	**1.46–29.27**	**9.37**	**1.71–51.29**	**6.35**	**1.42–28.45**

* Model 1: Multivariate OR adjusted for age, sex, BMI, Charlson comorbidity index, ethnicity, and ultraorthodox status. ** Model 2: Multivariate OR adjusted for age, sex, Charlson comorbidity index, ethnicity, and ultraorthodox status. Abbreviations: N, number; CI, confidence interval; OR, odds ratio. **Bold**: significant values.

**Table 3:** Demographics and characteristics of patients with vitiligo and comorbid malignant melanoma relative to the remaining patients with vitiligo.

	Vitiligo Without MM (N = 14,562)	Vitiligo Comorbid MM (N = 70)	*p*
	N (%)	N (%)	
Age, years * Mean, SD (range)	45.6, 17.3 (18–95.4)	61.8, 16.3 (18–92.3)	**<0.001**
Sex			
Males	7297 (50.1)	38 (54.2)	0.48
Ethnicity			
Jews	11,351 (77.9)	65 (92.8)	**0.003**
Arabs	3211 (22.1)	5 (7.2)	
Weight in Kg Mean (SD)	73.4 (16.3)	72.5 (14.0)	0.65
Missing	1763 (12.1)	2 (4)	
Height in meters, mean (SD)	1.68 (0.1)	1.68 (0.1)	0.81
Missing	2309 (15.8)	6 (8.6)	
BMI (kg/m^2^) Mean (SD)	26.1 (5.1)	25.8 (4.2)	0.67
Missing	2317 (15.9)	6 (8.6)	
Charlson comorbidity index, median (range)	0.7 (0–13)	3.0 (2–8)	<0.001

* Age at the first date of follow-up. Abbreviations: N, number; BMI, body mass index; SD, standard deviation; MM, malignant melanoma. **Bold**: significant values.

## Data Availability

The data that support the findings of this study are available from the corresponding author, K.K., upon reasonable request.
